# Dependence of the Impact Response of Polyvinylidene Fluoride Sensors on Their Supporting Materials' Elasticity

**DOI:** 10.3390/s130708669

**Published:** 2013-07-05

**Authors:** Yunfang Jia, Xinjuan Chen, Qingshan Ni, Longhua Li, Cheng Ju

**Affiliations:** The College of Information Technical Science, Nankai University, Tianjin 300071, China; E-Mails: chenxin_juan@126.com (X.C.); nqsnankai@163.com (Q.N.); longhuasmile@163.com (L.L.); 15114574349@163.com (C.J.)

**Keywords:** PVDF, piezoelectric polymer, impact response, peak-to-peak voltage, recovery time

## Abstract

Polyvinylidene fluoride (PVDF) is popular sensing material because of its unique piezoelectric characteristics. In this work an impact sensor was prepared from a sandwiched structure PVDF film, and the related detection circuits were presented. The dependence of the PVDF sensors' response on the elasticity of the supporting materials was examined and discussed. Here two response indexes were discussed, which were the peak-to-peak voltage (V_pp_) and the recovery time. Firstly, falling impact experiments were executed on desk-supported PVDF sensors (100 μm PVDF film) using free falls of different weights from different heights. Then the same shock experiments were repeated on the same sensor, but changing the backstops to a sponge and rubber, respectively. On the desk, the values of V_pp_ were bigger than when the other two backstops were used; but the changes of the impact energy could not be reflected by the PVDF sensor when it was supported by a hard material. It was found that the biggest sensitivity of the voltage response (about 96.62 V/J) was obtained by the sponge-supported sensor; for the same sensor, when it was supported by rubber, the slope was 82.26 V/J. Moreover, the recovery time for the desk-supported sensor was almost constant, varying from 0.15 to 0.18 s, while for the same sensor supported by sponge or rubber, its recovery time changed with the shifting of the impact energy in the range of 0.02∼0.36 s, but no pattern could be found in the recovery-time characteristics.

## 1. Introduction

Polyvinylidene fluoride (PVDF) is reported to possess the properties of piezoelectricity, pyroelectricity and ferroelectricity [1–3]. Because of the PVDF film features of high sensitivity, wide frequency response, flexibility, cost effectiveness, ease of fabrication and light weight, it has been a popular sensing material because of its unique piezoelectric characteristics, though many other kinds of piezoelectric polymers have been proposed [4].

For examples, deformation-caused piezoelectric sensing was induced by adhering PVDF sensors onto the surface of a wind turbine blade, allowing the damage to the blade to be monitored [5]. A novel acoustic sensor was developed using microsized PVDF pillars which were arrayed and sandwiched between two electrodes [6]. In the field of ultrasonic range sensors, both the transmitter and receiver could be built from separate curved PVDF transducers or a single one [7], and the ultrasonic properties of the large-area PVDF film were characterized and compared with PZT for the purpose of evaluating its performance in ultrasonic sensing [8]. In robotic applications, PVDF touch sensors were good candidates [9], a novel touching sensor named Piezoelectric Oxide Semiconductor FET (POSFET) was proposed [10] and applied in human skin condition monitoring [11]. Based on the traditional PVDF touch sensor, a multilayered PVDF film was adopted to construct pain sensors [12], and identification of materials could be performed by monitoring the peak of PVDF sensors' output signal after touching target objects [13,14], and the detection of incident slippage and static friction was executed by PVDF tactile sensors with structured electrodes [15]. When compared with fragile ceramic piezoelectric materials, the native tenacity and flexibility of PVDF piezoelectric film make it an ideal candidate for shock sensors [16]. Voltage signals is response to impacts could be examined in a collision [17] or an explosion [18]. PVDF sensors' biomedical applications are new research hot-spots. By a build-in POSFET PVDF sensors on the inside wall of the grasper, the pressure generated by the endoscope grasper on tissues could be monitored [19]. The same idea was also applied in lithotrities surgery [20] and catheters [21]. The intensity of shock waves generated in the surgery was fed back by a PVDF shock-wave hydrophone to guarantee the destruction of stones and the safety of benign tissues [20], and pressure could be determined in real time flow measurements [21]. Physiological pulse signals could be measured by a PVDF pressure sensor [22]; its high sensitivity was evidenced by its comparison with an Electro Mechanical Film (EMFi) device [23].

It is found in the limited review that the emphases of the reported studies are mainly focused on the preparation of PVDF piezoelectric polymer films in the early stage [1–4] and the exploration of PVDF related applications in the past twenty years [5–22]. It is accepted that the response of a PVDF senor is influenced by many conditions, such as the ambient temperature [24], the smoothness of the touched object [11] and the structure of electrodes [15]. It is also noted that the sandwiched structure is adopted as the sensing element in almost all of the referenced reports. In this structure the front-side is always used as the sensing surface, and much attention has been paid to what kind of response signals could be obtained by PVDF sensors when their sensing surfaces suffered external pressure [12,15,21], friction [15], deformation [5], impact with different energies [16–18,20] or were touched with different materials [13,14], *etc.*, while, for their back side there are very few concerns, as we know. Different backstops were used in different reports, such as the aluminum cantilever [8], plastics [9], silicon wafers [21], polydimethylsiloxane (PDMS) [22] and rubber [23], but the supporting materials' influence on PVDF sensors' response characteristics were seldom mentioned.

In this work, various backstops' influences on PVDF sensors' response to a falling shock are measured. The temporal voltage response signals for the same PVDF sensor are measured under three different supporting conditions, which are using a desk, rubber and a sponge as supporting materials, in order of increasing elasticity Two characteristics are discussed here, which are the peak-to-peak voltage (Vpp) of the response signals and the recovery time after suffering an impact. The qualitative analysis of their variation induced by backstops was presented as the coordinates of Vpp *vs.* the impact energy and the recovery time vs. the impact energy. The peak of the temporal response curves was suitable for a soft-supported PVDF sensor, while for rigid-supported ones, the changes of impact energy could be reflected by the areas under the response peaks of the temporal response curves, not the peaks' actual value.

## 2. Experimental Section

### 2.1. Materials

**Figure 1.**
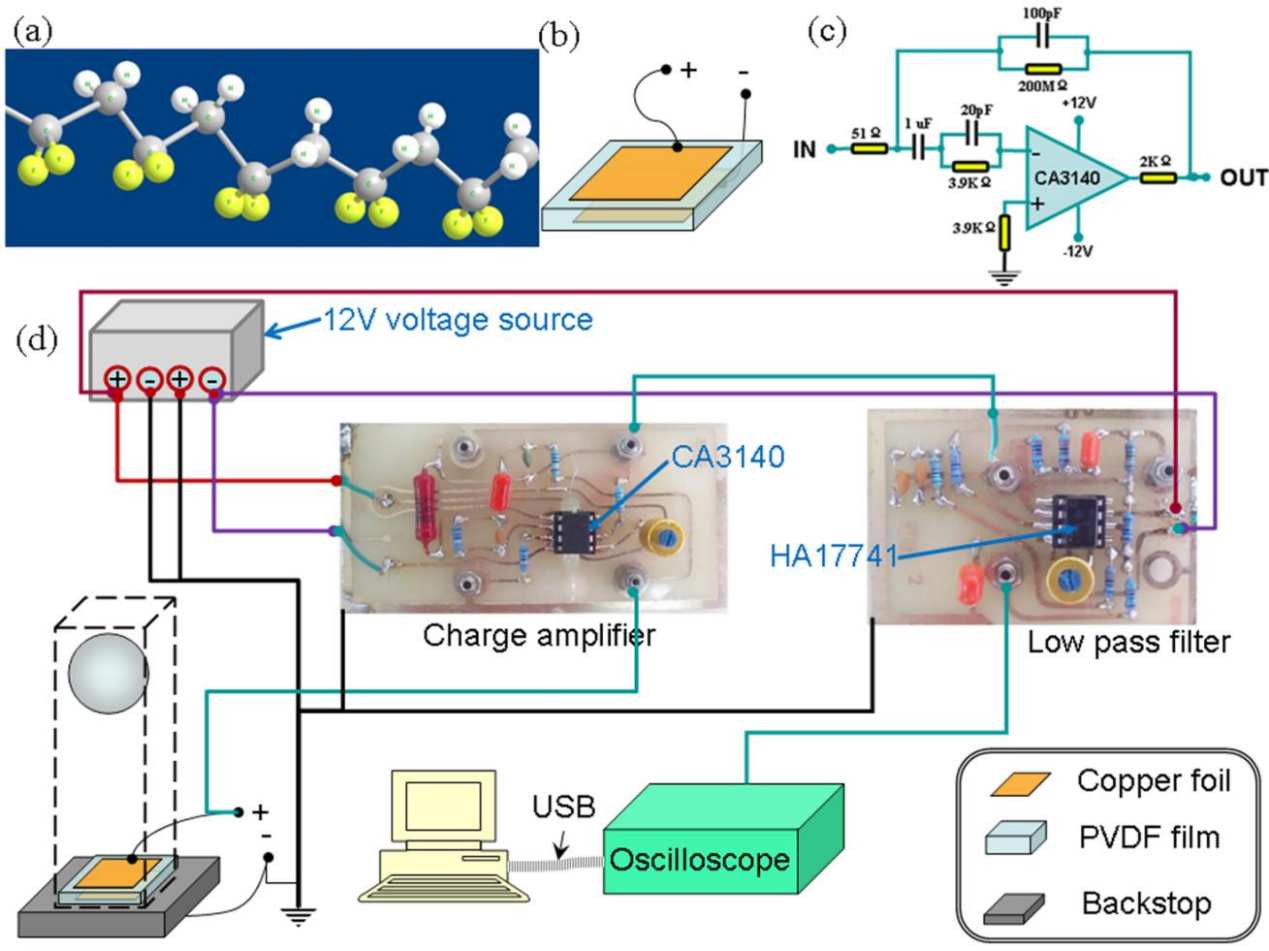
Sketch maps for the PVDF sensor and the related detection system: (**a**) the molecular structure of PVDF; (**b**) the sandwiched structure of PVDF sensor; (**c**) the principle circuit of the charge amplifier used to transform piezoelectric charges generated by PVDF sensor to a voltage signal; (**d**) the outline for the experimental setup and the detection system.

The PVDF piezoelectric film was purchased from Jinzhou KeXin Eectronic Materials Co. Ltd, (Jinzhou, China). The purchased sheet had been already stretched and poled. The type of PVDF film was KXP02, the thickness was 100 μm. The polarized polymer material had a zigzag molecular structure which as sketched in Figure 1(a). The atoms of fluorine and hydrogen were separated in the two sides of the carbon chain. There were spontaneous dipoles in this structure. After polarization PVDF could exhibit piezoelectric properties, and the charge coefficient, d_31_, d_32_ and d_33_ were 17 ± 1, 5∼6 and 21 ± 1 pico-Coulomb per Newton (pC/N), respectively.

Copper foil (DongGuan FeiYing Electronic Material Co. Ltd., DongGuan, China) was used as the electrode material. The thickness was 0.02–0.05 mm and there was a conducting resin on one side of the foil.

A charge amplifier was constructed to transform the sensing charge generated by PVDF into an output voltage signal. The principal circuit and a photo of the charge amplifier are given in Figure 1(c,d), respectively. Here CA 3140 was an integrated circuit operational amplifier (Harris Semiconductor Melbourne, Florida, USA), which was used to transfer the piezoelectric charges generated by the PVDF sensor. As shown in Figure 1(c), the impact sensing charges were input from the IN port; by the used of the parallel resistor-capacitor (200 MΩ and 100 pF) in the negative feedback loop of CA 3140, an impact-sensing voltage signal could be output from the OUT port. Furthermore, a low pass filter was used to screen out the ultimate voltage signal.

### 2.2. Experiments

The sensors were prepared based on PVDF films and copper foil with a sandwiched structure, as outlined in Figure 1(b). First two out-leads were soldered on tailored copper foils, and the Cu foils were transmitted and conglutinated onto the surfaces of PVDF film by the conducting resin on the copper foils. Second, the PVDF sensors were laid on different backstops with flat surfaces, which were desk, rubber and sponge. Third, a falling path was formed with hardboard to guarantee the impacting force happened exactly on the PVDF sensors. Here, two kinds of weights, which were 20 and 50 g, were tested; the weights fell down freely from three different heights, 10, 15, 20 cm.

### 2.3. Detection System and Equipments

The detection system was depicted in Figure 1(d) and the principal circuit for the charge amplifier was presented in Figure 1(c). The charges generated by the PVDF sensors' piezoelectricity were introduced into the charge amplifier, through the capacitor in Figure 1(c) the charges' variation of the PVDF sensor could be transformed and screened out as a voltage signal. Finally, the impacting sensitive voltage signals were displayed by a TDS2022B oscilloscope (Tektronix Inc. Beaverton, OR, USA), and the data were collected by a computer through a USB port and analyzed by Origin Lab 8.1 (Origin Lab Corporation, Northampton, MA, USA).

## 3. Results and Discussion

### 3.1. PVDF Sensors' Voltage Response Results When Supported by the Desk

**Figure 2.**
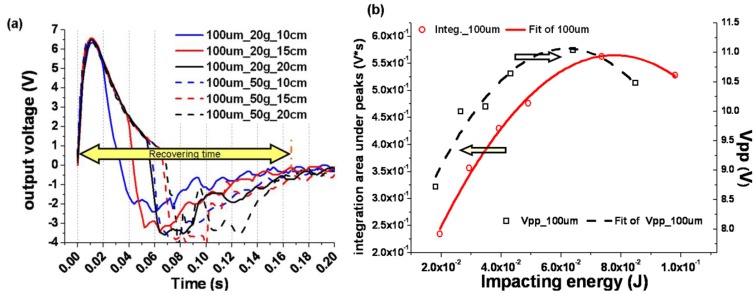
Response of PVDF sensor on the desk: (**a**) temporal response curves for the sensors prepared from 100 μm PVDF film when the weights were 50, 20 g and the falling height was changed from 10 to 20 cm; (**b**) the analysis of peaks' area and the peak-to-peak voltage (V_pp_) *vs.* the impacting energy.

The shock response of the PVDF sensor was measured on the desk; the temporal voltage response curves are presented in Figure 2(a). The falling impact conditions are adjusted according to Table 1. That means higher impacting energy could be obtained when the heavier object fell from the higher position. The impact energies were estimated and are listed in Table 1, by assuming all the mechanical potential energy was transformed to impact energy. To contrast their response characteristics under different conditions, the collected voltage signals were moved along the x-axis to aim at the same zero time.

The main explanation of the results is that the intensity of polarization in the PVDF film will be changed when it suffers a falling impact. To maintain the electric neutrality, it will release or capture charges from outside circuits as given in Figure 1(c). The quantity of these charges is measured and transformed into a voltage signal by the measuring circuits, given in Figure 1(c,d).

In Figure 2(b), the abscissa is the impact energy; the approximate values are given in Table 1. Similar curve shapes for these two analyzed items can be found in Figure 2(b). Since the area was the product of Vpp and the recovery time, so it was reasonable to deduce that the difference of the recovery time under different impacting conditions was tiny. Approximately calculations indicated that the recovery time for the desk supported sensor was about 0.15∼0.18 s.

By comparing the horizontally moved curves in Figure 2(a), it was found that: (1) under different impact conditions the positive peaks' amplitudes were not obviously changed, while the negative peaks were lowered, so the value of V_pp_ must be changed by the increasing impact energy, as shown by the right y-axis in Figure 2(b). (2) After the impact, the response voltages would return to their original value. In this progress there was a recovery time, a sample of which was depicted as a yellow double arrowhead line for the dashed black line in Figure 2(a), but it seemed that the variation of their recovery time was very small. Furthermore, V_pp_ and the integration areas under peaks were calculated by MATLAB, and their relation with the changing impacting energy was plotted in Figure 2(b). Here V_pp_ means the maximal output voltage minus the minimum output voltage, and integration areas were the areas under the curves from the highest to the lowest. These two items were plotted in a double- y-axis coordinates, as shown in Figure 2(b).

### 3.2. Results of the Elastically Supported PVDF Sensor

**Figure 3.**
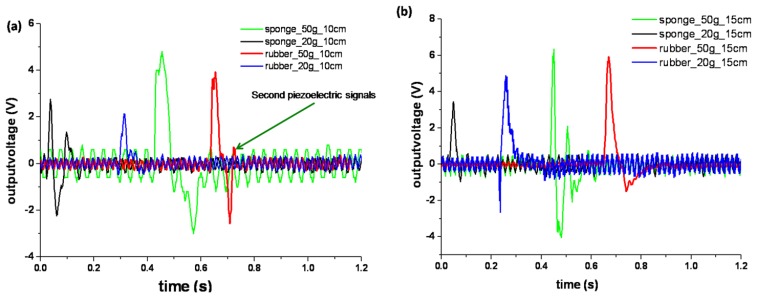

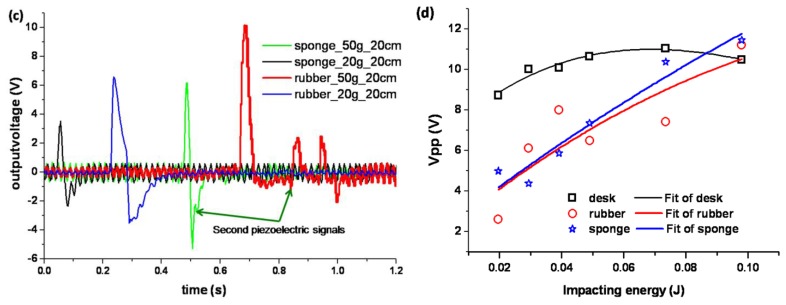
Response of PVDF sensors on sponge and rubber. (**a**-**c**) temporal response curves for the PVDF sensor prepared from 100 μm PVDF film, the weights were 50, 20 g, and the falling height was 10, 15, 20 cm; (**d**) the analysis of the peak-to-peak voltage (Vpp) *vs.* the impact energy for the same sensor prepared from 100 μm PVDF film.

After changing the backstops, the experiments mentioned above were repeated on the 100 μm PVDF sensor used in Figure 2. Here two kinds of supporting materials were tested, which were sponge and rubber. The temporal response curves were plotted in Figure 3(a-c). The values of Vpp for all the figures in Figure 3(a-c), and Figure 2(a) are analyzed and plotted in Figure 3(d).

It was found from Figure 3(a), that the response curves' vibration of the sponge supported sensor was stronger than the rubber supported one under the same impact conditions. We deduced that the reason might lie in the elasticity of the supporting materials. The elasticity of sponge was bigger than that of rubber, so it offered a buffering action to PVDF film sensor and made it suffer a second elastic force after undergoing the impact, and this force could cause a second-piezoelectric-signal which was labeled in Figure 3(a,c), while for the rubber supported one, this signal was relatively small. This deduction could be supported by further comparison with Figure 2(a). On the hard material (the wooden desk) with almost no elasticity, no second-piezoelectric-signal was detected.

Another result that could be obtained in Figure 3(a–c) was that Vpp changed with the increasing impacting energy. Their relationship was proposed in Figure 3(d). The ascending curves in Figure 3(b) indicated that with the increase in impact energies, the values of Vpp were enhanced, but the slopes of these curves were obviously different. Desk-supported sensors exhibited smaller V_pp_ variation than rubber and sponge supported ones. For the same sensor (100 μm), the biggest curves' slope of Vpp *v*s. impacting energy was displayed by the sponge-supported one, the slope of which was 96.62 V/J., while for the same sensor, when it was supported by rubber, the slope was 82.255 V/J; and when it was supported by the desk the voltage response saturated quickly.

### 3.3. Discussion

According to the experiments mentioned above, PVDF sensors could respond to a suffered impact and transform the mechanical energy into an electronic signal, but there were differences in their response signals when the sensors' supporting materials were changed.

Firstly, the impact power could be expressed in the value of the peak-to-peak voltage, so the V_pp_ *vs.* impact energy curve was chosen as the response curve. When the impact energy was in the range of 0.02∼0.08 J, the bigger V_pp_ values could be transduced by a hard supported sensors (like the desk used in this work) than by the sponge or rubber supported ones, but the difference of impact energy could not be discriminated by the desk supported sensor, which was shown by the black and purple lines in Figure 3(d), and the quantitative detection could be realized better by the sponge supported sensor than the rub ber or desk supported one, whose sensitivities were given in above subsection.

**Figure 4.**
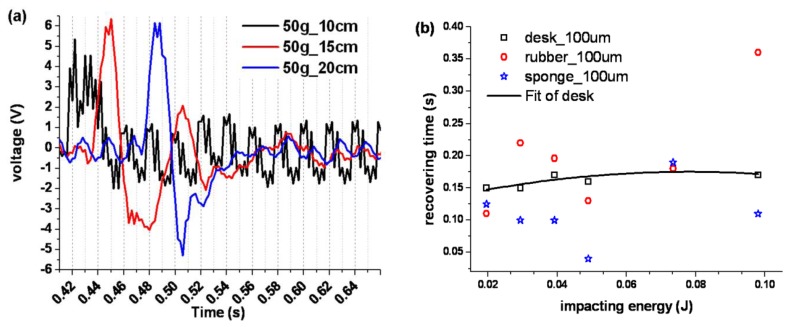
Comparison of the recovery time of the PVDF sensor after suffering impact, when it was supported by three backstops which were desk, rubber and sponge, respectively. (**a**) The enlarged temporal response curves of the sponge supported sensor after being impacted by 50 g weight from different height, 10, 15 and 20 cm. (**b**) The data of the PVDF sensor's recovery time in the coordinates of recovery-time *vs.* impact-energy, which were extracted from Figure 2(a) and Figure 3(a).

Secondly, the recovery time between differently supported sensors also varied. The green and black curves in Figure 3(a–c) represent the sponge supported sensor's response signals under three different conditions. These solid curves were magnified in the time range of 0.41∼0.66 s here, as shown in Figure 3(a), to observe the sponge supported PVDF sensor's temporal response characteristics. It was deduced according to the black solid curve in Figure 4(a), that the sensor might endure a sympathetic vibration after it suffered a falling impact induced by a 50 g weight dropped from a 10 cm height, since the amplitude output voltage was enhanced after suffering the impact and could not recover. Furthermore, the response time data was extracted and plotted under recovery-time *vs.* impacting-energy coordinates, shown in Figure 4(b). As given in the explanation of Figure 2(a), the recovery time for desk supported sensors was about 0.15 to 0.18 s. So the characteristic curve about recovering time for the desk supported sensor was nearly flat as shown by the fitted curve in Figure 4(b), but for the sponge and rubber supported sensor the recovery time was up-and-down and no order could be found.

Thirdly, comparisons between Figure 2(a), and Figure 3(a–c) indicate that, for the same sensor (100 μm), when the supporting materials are changed from desk, rubber, to sponge, more obvious second piezoelectric signals could be observed. This phenomenon was coincident with the elasticity of these three materials.

## 4. Conclusions

The supporting materials' influence on a PVDF sensor's impact response was tested and discussed in this work. Three kinds of supporting materials were examined, which were a desk, rubber and sponge, as examples of three kinds of rigidity or elasticity. There were obvious response signals for the PVDF sensor supported by all these materials. Their response characteristics on different supporting materials could be evaluated by the curves of V_pp_ *vs.* the impact-energy. The desk supported PVDF sensor could convert the impact energy into a relatively bigger V_pp_ than the same sensor supported by rubber or sponge, but the desk supported sensor was responsive enough to measure the impact intensity, because the values of V_pp_ were almost constant when the impact energies were changed. Combined with the discussion about the recovery time, it was summarized that the rigidly supported PVDF sensor could transform the impact to a relatively higher voltage signal (V_pp_) in a constant time; it would be a choice for a shock controlled switch. Impact intensity measurements could be executed on the softly supported PVDF sensor, like sponge in this work.

## Figures and Tables

**Table 1.** The approximate impact energy.

**Mass (g)**	**Height (cm)**	**Impact energy (J)**
20	10	0.0196
20	15	0.0294
20	20	0.0392
50	10	0.049
50	15	0.0735
50	20	0.098
